# Racial disparity in taxane‐induced neutropenia among cancer patients

**DOI:** 10.1002/cam4.4181

**Published:** 2021-09-21

**Authors:** Neil S. Zheng, Fei Wang, Rajiv Agarwal, Robert J. Carroll, Wei‐Qi Wei, Jordan Berlin, Xiao‐Ou Shu

**Affiliations:** ^1^ Department of Biomedical Informatics Vanderbilt University Medical Center Nashville TN USA; ^2^ Division of Epidemiology Department of Medicine Vanderbilt‐Ingram Cancer Center Vanderbilt University Medical Center Nashville TN USA; ^3^ Department of Breast Surgery The Second Hospital Cheeloo College of Medicine Shandong University Jinan Shandong People’s Republic of China; ^4^ Division of Hematology/Oncology Department of Medicine Vanderbilt‐Ingram Cancer Center Vanderbilt University Medical Center Nashville TN USA

**Keywords:** healthcare disparities, neoplasms, neutropenia, race

## Abstract

**Background:**

Large interindividual variations have been reported in chemotherapy‐induced toxicities. Little is known whether racial disparities exist in neutropenia associated with taxanes.

**Methods:**

Patients with a diagnosis of primary cancer who underwent chemotherapy with taxanes were identified from Vanderbilt University Medical Center's Synthetic Derivative. Multinomial regression models were applied to evaluate odds ratios (ORs) and 95% confidence intervals (CIs) of neutropenia associated with race, with adjustments for demographic variables, baseline neutrophil count, chemotherapy‐related information, prior treatments, and cancer site.

**Results:**

A total of 3492 patients were included in the study. Compared with White patients, grade 2 or higher neutropenia was more frequently recorded among Black patients who received taxanes overall (42.2% vs. 32.7%, *p* < 0.001) or paclitaxel (43.0% vs. 36.7%, *p* < 0.001) but not among those who received docetaxel (32.0% vs. 30.2%, *p* = 0.821). After adjustments for multiple covariates, Black patients who received chemotherapy with any taxanes had significantly higher risk of grade 2 (OR = 1.53; 95% CI = 1.09–2.14) and grade 3 (OR = 1.91; 95% CI = 1.36–2.67) neutropenia but comparable risk of grade 4 neutropenia (OR = 1.19; 95% CI = 0.79–1.79). Similar association patterns were observed for Black patients who specifically received paclitaxel, but a null association was found for those treated with docetaxel.

**Conclusion:**

Black cancer patients treated with taxanes for any cancer had a higher risk of neutropenia compared with their White counterparts, especially those who received paclitaxel. More research is needed to understand the mechanism(s) underlying this racial disparity in order to enhance the delivery of patient‐centered oncology.

## Abstract 2

After accounting for baseline neutropenia count, patient characteristics and other treatments received, Black patients who received chemotherapy with taxanes had a greater risk for neutropenia compared with their White counterparts, especially for those who received paclitaxel. This is critically important for treatment planning and dose adjustments, expectation setting, and physician awareness for the potential for increased infections or febrile episodes during periods of neutropenia in Black patients. Our findings call for further investigations on this disparity, particularly in clinical trial settings, as well as on the underlying mechanisms.

## 1 INTRODUCTION

Cancer is a major public health issue worldwide and the second leading cause of death in the United States (U.S.).^1^ It is estimated that over 1.8 million new cancer cases were diagnosed, and approximately 600,000 patients died of cancer across the United States in 2020.^1^, ^2^ Cancer survival has been substantially improved over the past 30 years, partly due to advances in cancer therapeutics, such as immunotherapy, targeted molecular therapy, and chemotherapy.^1^, ^2^

Taxanes, including paclitaxel and docetaxel, are a main class of chemotherapeutic drugs that have been used to treat multiple cancers (e.g., cancers of the breast, lung, ovary, etc.) since the 1960s.^3^ Taxanes act, primarily, through binding to tubulin and inhibiting microtubule disassembly in a time‐ and concentration‐dependent manner.^4^ The most common taxane dose‐limiting toxicity is neutropenia (i.e., neutrophil count < 1.5 × 10^3^/µL), which may result in chemotherapy dose delays or dose reductions. Severe neutropenia (i.e., neutrophil count < 0.5 × 10^3^/µL) and febrile neutropenia (i.e., neutrophil count < 0.5 × 10^3^/µL and fever) range from 6.9% to 23% in incidence^5^, ^6^ and are associated with longer hospitalization stays, as well as increased morbidity, mortality, and treatment costs.^7^

Large interindividual variations, including differences arising from race, have been observed in toxicities associated with chemotherapy.^8^ Chemotherapy‐induced toxicities, including neutropenia, may be a result of exposure to a specific drug, the dose‐density received, as well as each individual's underlying sensitivity to chemotherapy and susceptibility for developing toxicity.^9^ Understanding the factors related to interindividual variations in chemotherapy‐induced toxicities may facilitate the delivery of more personalized treatment and improve treatment and patient‐reported outcomes, such as quality of life. Of note, it has been previously described that neutropenia may be a common, albeit considered benign, phenomenon among healthy African Americans. This observation may necessitate chemotherapy dose modifications for safety, and thereby may contribute to widely reported racial/ethnic disparities in survival post‐cancer diagnosis.^10^ However, less is known whether race impacts the prevalence of neutropenia after receiving chemotherapy with taxanes. Because racial/ethnic minorities are historically underrepresented in clinical trials,^11^ this question has not been previously addressed in clinical trial settings.

To fill this knowledge gap, we utilized electronic health record (EHR) data from Vanderbilt University Medical Center (VUMC) to evaluate the influence of race on the risk of taxane‐induced neutropenia among patients diagnosed with a primary cancer.

## 2 METHODS

### 2.1 Study population

We identified 3,492 patients who were diagnosed with a primary cancer and received taxane (i.e., paclitaxel or docetaxel) treatment from VUMC’s Synthetic Derivative (SD), a data repository containing deidentified EHRs for over 3 million individuals, with records dating back to January 1993.^12^ This study was approved by the institutional review boards at VUMC.

### 2.2 Study variables and covariate assessment

We extracted taxane treatment information from medication administration records, which included details for each infusion that occurred at VUMC. These semi‐structured records included date and time of administration, type of taxane––identified by keywords “PACLITAXEL” and “DOCETAXEL”––and administered dose. Using this information, we derived overall taxane treatment start date, mean dosage, mean treatment duration (months), and number of treatment cycles. We focused on the first taxane treatment course for each individual. For cancer type, we determined the most recent diagnosis code prior to cancer treatment and manually grouped diagnosis codes by anatomical site. Cancer stage data were retrieved from the VUMC North American Association Central Cancer Registry (NAACR), where available.

Demographic information, including self‐reported race, age at first taxane treatment, sex, most recent body mass index (BMI, weight in kilograms/height in center meter^2^) measurement prior to the first taxane treatment, and ever smoking status, was retrieved from EHRs. Age at first taxane treatment was categorized into <55, 55 to <65, 65 to <75, ≥75. We also categorized BMI into underweight (BMI < 18.5), normal weight (BMI ≥ 18.5 to <25), overweight (BMI ≥ 25 to <30), obese (BMI = 30 to <40), and morbidly obese (BMI ≥ 40).

Neutrophil counts and measurement dates were extracted from the SD “measurement” table, which contained laboratory results for each individual, using the Logical Observation Identifiers Names and Codes (LOINC) code 3013650. LOINC is a standardized terminology for medical laboratory observations. Baseline neutrophil count (10^3^/µL) was estimated by taking the most recent neutrophil count prior to the first taxane treatment. Using keyword matches, we also created an indicator variable for prophylactic granulocyte colony‐stimulating factor (GCSF) treatment (namely filgrastim, pegfilgrastim, and sargramostim) between the month prior to the first taxane treatment and the first date of neutropenia onset. Prophylactic GCSF treatment has been shown to reduce the risk and severity of neutropenia.^13^ We additionally assessed several other covariates including prior chemotherapy and co‐chemotherapy via keyword matches, as well as prior radiotherapy using Current Procedural Terminology codes, standardized codes used to report medical services and procedures. These factors have been previously implicated as risk factors for neutropenia risk.^14^ All keywords and codes used to identify study variables are documented in Table S1.

### 2.3 Study endpoint

The primary study endpoint was any occurrence of neutropenia during the individual's first taxane treatment course. For each individual, we categorized measured neutrophil counts during their first taxane treatment course into neutropenia grades, following guidelines outlined by the US National Cancer Institute's Common Terminology Criteria for Adverse Events, v5.0^15^: grade 2 neutropenia (neutrophil count = 1.0 to <1.5 × 10^3^/µL), grade 3 neutropenia (neutrophil count = 0.5 to <1.0 × 10^3^/µL), and grade 4 neutropenia (neutrophil count <0.5 × 10^3^/µL).

### 2.4 Statistical analysis

Pearson *χ* ^2^ tests for categorical variables and Student *t*‐tests for continuous variables were performed to compare the characteristics of patients who received taxane treatment by self‐reported race.

Multinomial regression models were applied to evaluate odds ratios (ORs) and 95% confidence intervals (CIs) of neutropenia associated with self‐reported race and other demographic variables of interest (age at first treatment, sex, BMI, and ever smoking status). All variables of interest were included in the multinomial analysis with adjustments for mean taxane treatment dose, number of taxane treatment cycles, base neutrophil count, prior platinum treatment, prior radiotherapy, and cancer site. All statistical tests were based on two‐tailed probability and a significance level set at alpha (α) less than 0.05. Statistical analyses were performed in R v.3.6.1.

## 3 RESULTS

Selected characteristics of the 3,492 study participants who received chemotherapy with taxanes are shown in Table 1. Chemotherapy with paclitaxel was more frequently used than docetaxel (76.0% vs. 24.0%) among our study population. Overall, 22.3% of study participants received taxane treatment in combination with other chemotherapy agents. A majority of study participants (93.6%) received other chemotherapy agents prior to their first taxane treatment, and 23.6% of participants received radiotherapy prior to taxane treatment. In comparison to White patients, a higher proportion of Black patients received docetaxel (29.0% vs. 22.8%; *p* = 0.008). Black patients were more likely to receive taxanes concurrently with other chemotherapy agents (27.7% vs. 21.7%; *p* = 0.009) but were less likely to have had prior chemotherapy with platinum (59.2% vs. 72.0%; *p* < 0.001) or prior radiotherapy (18.9% vs. 25.0%; *p* = 0.010). There were no significant differences in mean treatment dose or taxane treatment duration between the two self‐identified racial groups.

**TABLE 1:** Characteristics of study participants stratified by self‐reported race

	All patients (N = 3492)	White patients (N = 3019)	Black patients (N = 365)	*p* value^a^
Age (years) at first treatment, Mean ± SD	59.2 ± 12.6	59.6 ± 12.4	56.7 ± 12.5	1.82 × 10^−5^
Age categorical, N (%)				0.003
<55	1166 (33.4)	966 (32.0)	144 (39.5)	
55 to <65	1059 (30.3)	920 (30.5)	118 (32.3)	
65 to <75	905 (25.9)	809 (26.8)	77 (21.1)	
≥75	362 (10.4)	324 (10.7)	26 (7.1)	
Sex, N (%)				3.68 × 10^−8^
Female	2046 (58.6)	1713 (56.7)	262 (71.8)	
Male	1466 (41.4)	1306 (43.3)	103 (28.2)	
Taxane type (%)				0.008
Docetaxel	838 (24.0)	689 (22.8)	106 (29.0)	
Paclitaxel	2654 (76.0)	2330 (77.2)	259 (71.0)	
Mean taxane treatment duration (months) ± SD	2.9 ± 4.7	2.9 ± 4.9	2.7 ± 3.2	0.380
BMI, Mean ± SD	28.2 ± 6.7	28.1 ± 6.4	30.2 ± 8.3	4.59 × 10^−9^
BMI categorical, N (%)				2.23 × 10^−8^
<18.5	113 (3.2)	94 (3.1)	13 (3.6)	
18.5 to <25	1093 (31.3)	958 (31.7)	89 (24.4)	
25 to <30	1112 (31.8)	987 (32.7)	96 (26.3)	
30 to <35	977 (28.0)	830 (27.5)	124 (34.0)	
≥40	197 (5.7)	150 (5.0)	43 (11.8)	
Ever smoke, N (%)	1587 (45.4)	1415 (46.9)	141 (38.6)	0.006
Cancer type, N (%) ^b^				4.16 × 10^−8^
Breast	1149 (32.9)	930 (30.8)	173 (47.4)	
Head & Neck	789 (22.6)	710 (23.5)	53 (14.5)	
Lung & Other Respiratory	624 (17.9)	559 (18.5)	50 (13.7)	
Female Genital & Reproductive	215 (6.2)	181 (6.0)	27 (7.4)	
Gastrointestinal	185 (5.3)	165 (5.5)	18 (4.9)	
Unknown	135 (3.9)	114 (3.8)	17 (4.7)	
Cancer stage, N (%) ^c^				0.0407
I	398 (11.4)	328 (10.9)	56 (15.3)	
II	472 (13.5)	405 (13.4)	46 (12.6)	
III	484 (13.9)	415 (13.7)	55 (15.1)	
IV	788 (22.6)	698 (23.1)	66 (18.1)	
Unknown	1350 (38.7)	1173 (38.9)	142 (38.9)	
Mean baseline neutrophil count (10^3^/µL) ± SD	5.6 ± 3.0	5.7 ± 3.0	5.1 ± 3.1	0.001
Mean treatment dose (mg/m^2^) ± SD	81.0 ± 45.0	80.5 ± 45.8	84.7 ± 40.1	0.094
Prophylactic GCSF, N (%)	271 (7.8)	220 (7.3)	42 (11.5)	0.004
Co‐chemotherapy, N (%)	777 (22.3)	654 (21.7)	101 (27.7)	0.009
Platinum, N (%)	687 (19.7)	583 (19.3)	85 (23.3)	0.071
Prior chemotherapy, N (%)	3270 (93.6)	2833 (93.8)	339 (92.9)	0.474
Platinum, N (%)	2466 (70.6)	2175 (72.0)	216 (59.2)	3.42 × 10^−7^
Prior radiotherapy, N (%)	833 (23.9)	755 (25.0)	69 (18.9)	0.010

^a^ *p* values derived from Pearson's *χ* ^2^ tests for independence for the categorical variables and Student *t* tests for the continuous variables comparing self‐reported race.

^b^ Top five cancer types are listed. Cancer types were determined using most recent diagnosis code to start of taxane treatment. Patients without diagnosis codes were grouped into “Unknown.”

^c^ Cancer stage was determined from VUMC NAACCR data, which was only available for patients diagnosed at VUMC.

Among all the study participants, breast cancer was the most common cancer treated with taxanes, followed by head & neck, lung & other respiratory, female genital & reproductive, and gastrointestinal cancers. Compared with White patients, a higher proportion of Black patients received taxane treatment for breast cancer (47.4% vs. 30.8%), but a lower proportion of Black patients received taxanes for head & neck (14.5% vs. 23.5%) and lung & other respiratory (13.7% vs. 18.5%) cancers. Compared with Black patients, White patients tended to be older, have lower BMIs, and were more likely to have ever smoked. There were significantly higher proportions of females among Black patients receiving taxanes than their White counterparts (71.8% vs. 56.7%). At baseline, Black patients had significantly lower neutrophil counts, on average, compared with White patients (mean neutrophil count = 5.1 vs. 5.7; *p* = 0.001) and were more likely to receive prophylactic GCSF treatment (11.5% vs. 7.3%; *p* = 0.004).

Among all study participants receiving taxanes, 39.8% experienced neutropenia of grade 2 or above during their first taxane treatment course (Table 2). Compared with White patients, grade 2 or above neutropenia was more frequently recorded among Black patients who received taxane overall (42.2% vs. 32.7%; *p* < 0.001) or paclitaxel (43.0% vs. 36.7%; *p* < 0.001), but there were no significant differences among patients receiving docetaxel (32.0% vs. 30.2%; *p* = 0.821).

**TABLE 2:** Frequency of neutropenia stratified by self‐reported race

Taxane treatment	All patients (N = 3492)	White patients (N = 3019)	Black patients (N = 365)	*p* value^a^
Any taxane user (%)^b^				7.17 × 10^−4^
None	2103 (60.2)	1837 (60.8)	196 (53.7)	
Mild (Grade 2)	455 (13.0)	387 (12.8)	57 (15.6)	
Moderate (Grade 3)	396 (11.3)	322 (10.7)	62 (17.0)	
Severe (Grade 4)	323 (9.2)	277 (9.2)	35 (9.6)	
Missing	215 (6.2)	196 (6.5)	15 (4.1)	
Paclitaxel user (%)				1.39 × 10^−4^
None	1564 (58.9)	1394 (59.8)	130 (50.2)	
Mild (Grade 2)	396 (14.9)	339 (14.5)	49 (18.9)	
Moderate (Grade 3)	338 (12.7)	278 (11.9)	52 (20.1)	
Severe (Grade 4)	185 (7.0)	161 (6.9)	19 (7.3)	
Missing	171 (6.4)	158 (6.8)	9 (3.5)	
Docetaxel user (%)				0.821
None	539 (64.3)	443 (64.3)	66 (62.3)	
Mild (Grade 2)	59 (7.0)	48 (7.0)	8 (7.5)	
Moderate (Grade 3)	58 (6.9)	44 (6.4)	10 (9.4)	
Severe (Grade 4)	138 (16.5)	116 (16.8)	16 (15.1)	
Missing	44 (5.3)	38 (5.5)	6 (5.7)	

^a^ *p* values derived from Pearson's *χ* ^2^ tests for independence comparing self‐reported race.

^b^ Reported percentages are for treatment subcategories.

Findings for racial differences in neutropenia remained after adjustments for age, sex, BMI, smoking status, mean treatment dose, number of treatment cycles, baseline neutrophil count, prior platinum treatment, prior radiotherapy, and cancer site. Compared with White patients, Black patients who received chemotherapy with any taxanes had significantly higher risk of grade 2 (OR = 1.53; 95% CI = 1.09–2.14) and grade 3 neutropenia (OR = 1.91; 95% CI = 1.36–2.67) but a comparable risk of grade 4 neutropenia (OR = 1.19; 95% CI = 0.79–1.79; Table 3). Additionally, male patients had a significantly lower risk of grade 3 neutropenia (OR = 0.63; 95% CI = 0.46–0.86) than female patients, and patients with a BMI ≥40 kg/m^2^ showed a lower risk of grade 2 neutropenia (OR = 0.42; 95% CI = 0.23–0.76) compared with those with BMIs between 18.5 and <25 kg/m^2^.

**TABLE 3:** Race and other demographic factors associated with neutropenia among all taxane patients

	Mild (Grade 2) (N = 455)		Moderate (Grade 3) (N = 396)		Severe (Grade 4) (N = 323)		
	N^a^	OR (95% CI)^b^	N	OR (95% CI)	N	OR (95% CI)
Race						
White	387 / 2224	Reference	322 / 2159	Reference	277 / 2114	Reference
Black	57 / 253	1.53 (1.09–2.14)	62 / 258	1.91 (1.36–2.67)	35 / 231	1.19 (0.79–1.79)
Other	11 / 81	0.74 (0.38–1.44)	12 / 82	1.02 (0.53–1.94)	11 / 81	1.09 (0.55–2.17)
Age						
<55	154 / 853	Reference	138 / 837	Reference	100 / 799	Reference
55 to <65	133 / 804	0.89 (0.67–1.18)	113 / 784	0.81 (0.60–1.09)	88 / 759	0.93 (0.67–1.30)
65 to <75	123 / 659	1.16 (0.86–1.55)	98 / 634	0.94 (0.69–1.29)	93 / 629	1.12 (0.80–1.57)
≥75	45 / 242	1.08 (0.72–1.63)	47 / 244	1.19 (0.79–1.79)	42 / 239	1.23 (0.79–1.91)
Sex						
Female	281 / 1482	Reference	245 / 1446	Reference	192 / 1393	Reference
Male	174 / 1076	0.89 (0.65–1.23)	151 / 1053	0.63 (0.46–0.86)	131 / 1033	0.75 (0.53–1.06)
BMI						
18.5 to <25	153 / 803	Reference	129 / 779	Reference	100 / 750	Reference
<18.5	13 / 75	0.81 (0.39–1.69)	17 / 79	1.30 (0.68–2.48)	14 / 76	1.80 (0.91–3.57)
25 to <30	153 / 817	0.96 (0.73–1.25)	112 / 776	0.90 (0.67–1.21)	108 / 772	1.12 (0.82–1.53)
30 to <35	121 / 724	0.83 (0.63–1.10)	116 / 719	0.99 (0.74–1.33)	78 / 681	0.88 (0.63–1.24)
≥40	15 / 139	0.42 (0.23–0.76)	22 / 146	0.69 (0.41–1.18)	23 / 147	1.01 (0.59–1.72)
Ever smoke						
No	223 / 1220	Reference	200 / 1197	Reference	161 / 1158	Reference
Yes	208 / 1166	1.02 (0.80–1.30)	177 / 1135	0.87 (0.67–1.12)	151 / 1109	0.85 (0.65–1.13)

^a^ Reporting as counts of patients in category / sum of patients with no neutropenia and patients in category.

^b^ All variables included in the multinomial analysis with adjustments for mean treatment dose, number of treatment cycles, and base neutrophil count, prior platinum treatment, prior radiotherapy, cancer site, and all other variables included in the table.

When analyses were restricted to patients who received paclitaxel, similar association patterns and point estimates for neutropenia were observed for race, sex, and BMI (Table 4). In addition, patients aged ≥75 at first paclitaxel treatment had significantly higher risk of grade 4 neutropenia (OR = 1.95; 95% CI = 1.09–3.50) than younger patients (i.e., age <55).

**TABLE 4:** Race and other demographic factors associated with neutropenia among paclitaxel patients

	Grade 2 Neutropenia (N = 396)		Grade 3 Neutropenia (N = 338)		Grade 4 Neutropenia (N = 185)		
	N^a^	OR (95% CI)^b^	N	OR (95% CI)	N	OR (95% CI)
Race						
White	339 / 1733	Reference	278 / 1672	Reference	161 / 1555	Reference
Black	49 / 179	1.47 (1.01–2.15)	52 / 182	1.86 (1.27–2.72)	19 / 149	1.16 (0.66–2.04)
Other	8 / 48	0.77 (0.35–1.72)	8 / 48	0.90 (0.41–2.01)	5 / 45	1.00 (0.36–2.80)
Age						
<55	125 / 617	Reference	111 / 603	Reference	48 / 540	Reference
55 to <65	125 / 624	1.02 (0.75–1.38)	92 / 591	0.81 (0.58–1.12)	56 / 555	1.25 (0.79–1.99)
65 to <75	108 / 523	1.16 (0.84–1.60)	90 / 505	1.03 (0.73–1.45)	54 / 469	1.52 (0.95–2.43)
≥75	38 / 196	1.05 (0.68–1.64)	45 / 203	1.30 (0.84–2.01)	27 / 185	1.95 (1.09–3.50)
Sex						
Female	240 / 1051	Reference	208 / 1019	Reference	118 / 929	Reference
Male	156 / 909	0.83 (0.59–1.15)	130 / 883	0.59 (0.42–0.82)	67 / 820	0.71 (0.45–1.13)
BMI						
18.5 to <25	134 / 612	Reference	108 / 586	Reference	59 / 537	Reference
<18.5	12 / 62	0.78 (0.36–1.70)	14 / 64	1.23 (0.61–2.48)	10 / 60	2.07 (0.89–4.82)
25 to <30	132 / 632	0.93 (0.70–1.25)	99 / 599	0.91 (0.66–1.26)	56 / 556	0.98 (0.64–1.50)
30 to <35	105 / 549	0.78 (0.57–1.06)	97 / 541	0.91 (0.66–1.26)	47 / 491	0.84 (0.53–1.32)
≥40	13 / 105	0.37 (0.19–0.71)	20 / 112	0.65 (0.37–1.16)	13 / 105	0.78 (0.38–1.60)
Ever smoke						
No	186 / 857	Reference	167 / 838	Reference	85 / 756	Reference
Yes	192 / 980	1.04 (0.80–1.36)	159 / 947	0.89 (0.67–1.17)	93 / 881	0.97 (0.67–1.41)

^a^ Reporting as counts of patients in category / sum of patients with no neutropenia and patients in category.

^b^ All variables included in the multinomial analysis with adjustments for mean treatment dose, number of treatment cycles, and base neutrophil count, prior platinum treatment, prior radiotherapy, and cancer site.

A similar race neutropenia association pattern was also observed among patients who received chemotherapy with docetaxel, although the point estimates failed to reach statistical significance (Table 5). These analyses were based on a much smaller sample size. We found that, compared with patients aged <55 at first treatment, those aged between 55 and 64 had a lower risk of grade 2 neutropenia (OR = 0.33; 95% CI = 0.13–0.86), while those aged between 65 and 74 had a lower risk of grade 3 neutropenia (OR = 0.36; 95% CI = 0.13–0.97). Patients with BMIs between 25 and <30 showed a higher risk of grade 4 neutropenia (OR = 1.69; 95% CI = 1.01–2.83) compared with those with BMIs between 18.5 and <25.

**TABLE 5:** Race and other demographic factors associated with neutropenia among docetaxel patients

	Grade 2 Neutropenia (N = 59)		Grade 3 Neutropenia (N = 58)		Grade 4 Neutropenia (N = 138)		
	N^a^	OR (95% CI)^b^	N	OR (95% CI)	N	OR (95% CI)
Race						
White	48 / 491	Reference	44 / 487	Reference	116 / 559	Reference
Black	8 / 74	1.45 (0.60–3.51)	10 / 76	1.71 (0.75–3.93)	16 / 82	0.95 (0.50–1.80)
Other	3 / 33	0.83 (0.22–3.04)	4 / 34	1.49 (0.47–4.79)	6 / 36	0.78 (0.29–2.08)
Age						
<55	29 / 236	Reference	27 / 234	Reference	52 / 259	Reference
55 to <65	8 / 180	0.33 (0.13–0.86)	21 / 193	0.81 (0.39–1.68)	32 / 204	0.65 (0.38–1.13)
65 to <75	15 / 136	1.66 (0.75–3.69)	8 / 129	0.36 (0.13–0.97)	39 / 160	0.87 (0.48–1.56)
≥75	7 / 46	1.74 (0.49–6.12)	2 / 41	0.28 (0.05–1.44)	15 / 54	0.90 (0.39–2.04)
Sex						
Female	41 / 431	Reference	37 / 427	Reference	74 / 464	Reference
Male	18 / 167	2.80 (0.80–9.75)	21 / 170	0.92 (0.33–2.54)	64 / 213	1.23 (0.65–2.35)
BMI						
18.5 to <25	19 / 191	Reference	21 / 193	Reference	41 / 213	Reference
<18.5	1 / 13	1.04 (0.10–10.71)	3 / 15	1.53 (0.25–9.41)	4 / 16	1.85 (0.48–7.19)
25 to <30	21 / 185	0.91 (0.41–1.99)	13 / 177	0.84 (0.36–1.92)	52 / 216	1.69 (1.01–2.83)
30 to <35	16 / 175	1.03 (0.47–2.25)	19 / 178	1.45 (0.68–3.09)	31 / 190	1.29 (0.73–2.29)
≥40	2 / 34	0.66 (0.13–3.25)	2 / 34	0.73 (0.15–3.55)	10 / 42	2.32 (0.98–5.46)
Ever smoke						
False	37 / 363	Reference	33 / 359	Reference	76 / 402	Reference
True	16 / 186	0.97 (0.47–2.01)	18 / 188	0.88 (0.44–1.76)	58 / 228	0.94 (0.59–1.48)

^a^ Reporting as counts of patients in category / sum of patients with no neutropenia and patients in category.

^b^ All variables included in the multinomial analysis with adjustments for mean treatment dose, number of treatment cycles, and base neutrophil count, prior platinum treatment, prior radiotherapy, and cancer site.

## 4 DISCUSSION

In this single‐center study, we found that Black cancer patients who received chemotherapy with taxanes had a higher risk of grade 2 or grade 3 neutropenia compared with White patients, even after adjustments for baseline neutropenia count and other treatments received. This higher risk was mainly confined to patients who received chemotherapy with paclitaxel. No significant differences were found among those who received docetaxel.

Neutropenia is one of the most common side effects of chemotherapeutic agents, including taxanes.^5^, ^16^ Although it has been frequently reported that large interindividual variations exist in toxicity associated with anticancer therapy, data on racial and/or ethnic variations in chemotherapy‐induced neutropenia have been limited, and results varied across cancer sites or chemotherapy agents.^17^, ^18^, ^19^ To date, no studies have systematically evaluated racial differences in toxicity from taxane‐induced neutropenia. In our study, with nearly 3,500 cancer patients evaluated, Black patients who received chemotherapy with taxanes showed a higher risk of neutropenia than White patients. There are several possible explanations for this observed racial disparity. First, several studies, including our present study, have shown that, compared with White individuals, neutropenia, or lower neutrophil counts are more common even among healthy Black individuals.^10^ Thus, Black cancer patients may be more susceptible to chemotherapy‐induced neutropenia compared with their White counterparts. This concern may have already been recognized in clinical practice, as we observed that a higher proportion of Black patients received prophylactic GCSF when receiving taxanes. Second, Black individuals have lower smoking rates and typically smoke fewer cigarettes than White individuals,^20^, ^21^ as observed among our study population. While it has been previously reported that nonsmokers treated with paclitaxel, docetaxel, and other chemotherapy agents had a higher incidence of neutropenia in comparison with smokers,^22^ smoking was not significantly associated with neutropenia in our study.

Our study reveals that racial disparity in taxane‐induced neutropenia cannot be explained by the above described differences between White and Black patients alone. After accounting for baseline neutrophil count, smoking history, as well as other demographic‐ and treatment‐related information, this disparity persisted, indicating the possibility that other unidentified contributors exist. It is possible that the racial disparities reflect the effects of structural racism on health disparities in the United States.^23^ For instance, predominantly Black neighborhoods are disproportionately affected by a lack of nutritional infrastructure as a result of residential segregation.^24^ Nutritional deficiencies, such as vitamin B12 and folate deficiencies, have been associated with neutropenia.^25^ Further work is needed to better understand the impacts of structural racism on racial disparity in neutropenia in cancer patients. In addition, pharmacogenetic studies have shown that single nucleotide polymorphisms (SNPs) located in genes encoding key metabolic enzymes, such as drug transporters (e.g., ABCB1) and the cytochrome P450 family (e.g., CYP3A4, CYP3A5, and CYP2C8) are associated with taxane‐induced toxicities.^26^, ^27^ Different distributions of metabolic composite groups related to the above SNPs have been reported between Black and White individuals,^28^ which may also contribute to varied taxane sensitivity and toxicity.^29^ Although both paclitaxel and docetaxel share similar structures, anticancer mechanisms, and key metabolic enzymes (i.e., CYP3A and CYP2C8), a previous study has also shown that paclitaxel pharmacokinetics are more likely to be influenced by environmental and genetic factors.^30^ Our finding that racial disparity in taxane‐induced neutropenia was only observed among patients who received paclitaxel but not among those who received docetaxel, appears to support this pharmacogenomic hypothesis. However, we cannot rule out the possibility that the null association among patients who received docetaxel was a result of the smaller sample size. It is also worth mentioning that our study was a retrospective analysis of single‐center data. Future validation studies using multi‐center data, ideally within randomized clinical trials, are warranted. Nonetheless, our findings highlight the need to investigate potential contributors to the racial disparity in taxane‐induced neutropenia and improve evidence‐based precision medicine to manage this common side effect.

In addition to the observed racial variations in chemotherapy‐induced neutropenia, we also found variations associated with sex and BMI. In general, male or obese cancer patients had a lower risk of neutropenia than their counterparts, especially in terms of mild or moderate neutropenia. Our findings regarding BMI‐associated variations are in line with previous studies, which showed that patients with higher BMIs had reduced risk or rates of chemotherapy‐induced neutropenia.^31^ This phenomenon has been consistently observed across various cancer types and chemotherapy agents.^32^, ^33^, ^34^ Although the mechanisms behind the observed reduced neutropenia risk among obese cancer patients have not been well‐illuminated, one possible explanation is that obese patients are more likely to receive planned empirical dose reduction, also known as “dose capping,” of chemotherapy agents.^31^ Some studies have also indicated that pharmacokinetic profiles of chemotherapy agents may be altered among obese patients,^35^ although more research on pharmacokinetic parameters is warranted. On the other hand, results on sex‐disparity in chemotherapy‐induced neutropenia have been inconclusive. While one study reported that female patients were at higher risk of post‐chemotherapy toxicities, including neutropenia,^36^ others showed similar risk of neutropenia between male and female cancer patients.^19^, ^37^ However, cancer types and chemotherapy agents varied across these studies, and further research with a specific focus on chemotherapy with taxanes is needed.

To the best of our knowledge, this is the first study to systemically evaluate racial disparity in taxane‐induced graded neutropenia among patients with a primary cancer diagnosis. The results of our study were further strengthened by comprehensive adjustments for a wide range of factors, particularly baseline neutrophil count and other cancer treatments, as well as age, BMI, and smoking history. There are also some limitations of our study. First, we cannot rule out the potential selection bias inherited from the single‐center study design. Patients treated at VUMC, an academic tertiary care center, may have different demographic characteristics and significant co‐morbid conditions from those treated in primary health care facilities. It is also possible that some patients may have had treatment at other healthcare settings prior to VUMC, which may have biased the results. Second, information on treatment, occurrence of neutropenia, as well as self‐reported race was extracted from the medical records. For patients who were transferred from other hospitals, information on prior history of taxane use was unavailable. Thus, misclassifications could not be ruled out, which would likely bias our study results toward null. Third, despite the relatively large sample size overall, we were not able to evaluate cancer‐specific race neutropenia associations due to the small sample sizes for individual cancer types. Moreover, discontinuation of treatment, comorbidity, detailed regimen (i.e., weekly or every 3 weeks), and post‐chemotherapy care (e.g., dietary quality, etc.), which may also be related to the occurrence of neutropenia, could not be accounted for due to the lack of relevant information. Finally, we could not evaluate racial disparity in febrile neutropenia, the most severe neutropenia, due to the lack of a reliable measurement.

In conclusion, after accounting for baseline neutropenia count, patient characteristics, and other treatments received, Black patients who received chemotherapy with taxanes had a greater risk for neutropenia compared with White patients, especially for those who received paclitaxel. This is critically important for treatment planning and dose adjustments, expectation setting, and physician awareness for the potential for increased infections or febrile episodes during periods of neutropenia in Black patients. Our findings call for further investigations on this disparity, particularly in clinical trial settings, as well as on the underlying mechanisms.

## CONFLICT OF INTEREST

The authors indicate no potential conflict of interest.

## AUTHORS CONTRIBUTION

NSZ, FW, and XOS conceived the study and developed the analytical plan; NSZ analyzed the data; NSZ and FW drafted the paper; RA and JB contributed to the study design; RC and WQW contributed to gathering the data; All authors critically reviewed and approved the paper.

## ETHICAL STATEMENT

This study used completely de‐identified data and was approved by the Institutional Review Board of Vanderbilt University Medical Center as non‐human subject research.

## Supporting information

Table S1

## Data Availability

Data available on request. The data underlying this article will be shared upon reasonable request to the corresponding author.
