# Supplementary material for: Racial disparity in taxane‐induced neutropenia among cancer patients
Source: Cancer Med. 2021 Sep 21;10(19):6767–76. doi: 10.1002/cam4.4181 (PMC8495275; doi:10.1002/cam4.4181)
Supplement: Supplementary file 1 — Table S1 [file CAM4-10-6767-s001.docx]

| **Supplementary Table 1. Keywords and codes used to identify study variables** | |
| --- | --- |
| **Study Variable** | **Keywords / Codes** |
| Taxanes | Paclitaxel  Docetaxel |
| Neutrophil count | LOINC code: 3013650 |
| Granulocyte colony-stimulating factor treatment | Filgrastim  Pegfilgrastim  Sagrgramostim |
| Chemotherapy (excluding taxanes) | Rubicin  Mitoxantrone  Vinorelbine  Eribulin  Ixabepilone  Platin  Fluorouracil (often abbreviated as 5-FU)  Capecitabine  Gemcitabine  Cytarabine  Cyclophosphamide  Etoposide  Irinotecan  Camptothecin  Trastuzumab  Pertuzumab  Bevacizumab  Ciclib |
| Radiotherapy | CPT codes:  77401-77402  77407  77412  77385-77386  77417  77387  77014  77520-77525  77422-77423  77371-77373  77600-77620  77778  77770  77772  0394T-0395T  77424-77425  77789  77750  77761-77763  77790 |
